# Development, Safety, Issues, and Challenges of the SARS-CoV-2 Vaccine

**DOI:** 10.3390/vaccines11030569

**Published:** 2023-03-01

**Authors:** Yasunari Matsuzaka, Ryu Yashiro

**Affiliations:** 1 Division of Molecular and Medical Genetics, Center for Gene and Cell Therapy, The Institute of Medical Science, University of Tokyo, Minato-ku 108-8639, Tokyo, Japan; 2 Administrative Section of Radiation Protection, National Institute of Neuroscience, National Center of Neurology and Psychiatry, Kodaira 187-8551, Tokyo, Japan; 3 Department of Infectious Diseases, Kyorin University School of Medicine, 6-20-2 Shinkawa, Mitaka-shi 181-8611, Tokyo, Japan

It has been reported that some mutant strains of the new coronavirus escape from neutralizing antibodies acquired by recoverees and vaccine recipients, in which the Omicron strain (B.1.1.529, BA strain) is resistant to therapeutic antibody formulations as well as neutralizing antibodies induced by two mRNA vaccinations [1]. Thus, a highly versatile vaccine that can respond to various mutant strains is expected in the future. There are some different types of viral vaccines: “live vaccines”, which use attenuated microorganisms; “inactivated vaccines”, which are produced by treating microorganisms with chemicals to eliminate infectivity or by purifying the proteins of microorganisms. In the case of a live vaccine, an actual viral infection is established, even if the toxicity is low. Thus, the production of neutralizing antibodies, humoral immunity, activation of cytotoxic T cells, and cellular immunity can occur. In other words, a strong immunity that can suppress viral infection is induced. Live vaccines are a powerful method; however, the establishment of attenuated virus strains takes a long time. In addition, the virulence of live vaccines can revert in the recipient’s body in rare cases. In contrast, inactivated vaccines are non-infectious and safe. In this case, the virus particles cannot be infected; therefore, they are recognized as foreign substances and transported to antigen-presenting cells (such as macrophages and dendritic cells) in which only humoral immunity is induced. Most viral infections occur locally on the mucous membranes. Therefore, if a vaccine can induce the production of IgG only in humoral immunity, antibodies cannot bind to the virus due to mucus obstruction, and infection cannot be suppressed.

In the case of influenza vaccines, embryonated chicken eggs (which are developing embryos) are inoculated with the virus, recovered, and inactivated to provide a large supply of the vaccine [2]. However, virus production using hatched chicken eggs has not been successful in the context of COVID-19. This is because the SARS-CoV-2 has different characteristics such as receptors, so unlike the influenza virus, it cannot multiply inside an egg. The egg-based vaccine, which takes six months to produce, could take too long, so the virus injected into the egg could mutate, thus rendering the vaccine less effective. In addition, vaccine production using the Vero cell line has been performed previously [3]. For this purpose, it is important to select an appropriate vaccine strain. On the other hand, mRNA vaccines and viral vector vaccines inject part of the genetic information that forms the basis for making viral proteins. Based on this information, part of the viral protein is produced in the human body, and antibodies against it are produced, thereby providing immunity to the virus. The advantages of these mRNA vaccines are as follows: no infectivity, no contamination with cell components, induction of cell-mediated immunity, no need for adjuvants, and relatively simple and inexpensive production [4,5]. The development and production of mRNA vaccines are relatively simple, scalable, and extremely rapid. mRNA technology is also attractive for the development of novel therapeutics in response to infectious disease outbreaks and pandemics, as it can shorten the time from development to clinical trial and approval. As mRNA is produced in vitro via an enzymatic process, in vitro synthesis does not require the removal of cellular or host cell proteins. Because this simplification of the manufacturing process allows the same reaction materials and vessels to be used for different targets, manufacturing facilities can switch to manufacturing mRNAs encoding new target proteins in a very short period with minimal process and formulation adaptations. The starting material for the production of mRNA-based vaccines is usually a plasmid DNA template (pDNA) that includes a DNA-dependent RNA polymerase promoter and sequences corresponding to the mRNA construct of interest [6]. Since the pDNA construct plays a central role, its design and purity are critical factors in optimizing the resulting mRNA. The preparation and purification of pDNA presents several challenges, such as the large size of nucleic acids, high viscosity, sensitivity to shear, and impurities similar to pDNA. Furthermore, research and development of vaccines based on self-replicating RNA obtained from alphaviruses are also underway [7,8]. When self-replicating RNA (which does not contain capsid proteins and therefore does not produce infectious viruses) is introduced into the cell, it expresses viral polymerase and initiates RNA replication. In addition, self-replicating RNA is replicated in a double-stranded state. Thus, it activates receptors that recognize double-stranded RNA and induces an immune response. Because replicating RNA self-replicates inside the cell, it is effective at low doses.

Although this mRNA vaccine utilizes protein translational machinery, artificial mRNA produced by in vitro transcription is introduced into cells using lipid nanoparticles or electroporation. The artificial mRNA that enters the cytoplasm binds to the ribosomes, and proteins are produced based on this information. However, for artificial mRNAs to be efficiently translated in vivo, recognition by the innate immune system must be avoided. Unlike the adaptive immune system, which recognizes only specific foreign molecules named pathogen-associated molecular patterns (PAMPs), the innate immune system functions by recognizing “common patterns among pathogens” and “endogenous molecules released when cells are damaged”, named damage-associated molecular patterns (DAMPs). Both patterns trigger downstream immune responses by binding to pattern recognition receptors (PRPs). Toll-like receptors 7 and 8 (TLR7/TLR8) sense pathogen-derived single-stranded RNA, and activation of TLR8 is triggered by the coordinated effects of single-stranded RNA and its degradation product, uridine [9]. Activation of the innate immune system causes an inflammatory response, suppression of mRNA translation, and degradation of mRNA. To avoid recognition of the administered mRNA by the innate immune system, the mRNA sequence was modified or chemically modified to add a cap structure [10]. In terms of sequences, uridines should be removed from the final mRNA sequence, as uridine-rich sequences elicit an innate immune response. In this case, using synonymous codons that encode the same amino acids and replacing codons containing uridine in the sequence with synonymous codons that do not contain uridine, the immunogenicity of mRNA can be suppressed while producing the same protein. Substitutions to modified nucleoside triphosphates are also effective (e.g., pseudo-uridine triphosphate, N1-methylpseudouridine triphosphate, and 5-methoxyuridine triphosphate, which evade detection by innate immunity) [11,12]. In addition, mature mRNAs produced in higher eukaryotes also have a 5′ cap structure called CAP-1, in which 7-methylguanosine (m7G) binds to the first nucleotide of the mRNA. Moreover, the 2′ ribose of the first nucleotide is methylated as a defense against innate immune responses, which enhances translation efficiency in vivo. The mRNAs with CAP-1 structures have low binding affinity for interferon-induced protein with tetratricopeptide repeats (IFIT), which recognizes differences in the cap structure of RNA and induces an innate immune response [13]. Additionally, mRNAs with CAP-1 structures avoid mRNA recognition by melanoma differentiation-associated 5 (MDA5), which recognizes long double-stranded RNA as a substrate [14]. However, because mRNA transcribed in vitro does not have a cap structure, it is necessary to add a cap structure during the manufacturing process to obtain an effective vaccine effect. In addition, regarding the difference in mechanism from conventional vaccines, once conventional vaccines such as live attenuated vaccines, inactivated vaccines, component vaccines, adjuvants, etc. are entered inside the body, the proteins and dead viruses are directly taken up by the antigen-presenting cells. Additionally, once the live attenuated vaccine is taken into the tissue cells of the inoculation site, the virus multiplies in the cells. The thing that came out from there is similarly recognized by antigen-presenting cells. The virus-derived peptides are presented as “foreign substances” (antigens), T cells and B cells react, and adaptive immune responses occur in a few weeks and are thought to continue for several years. On the other hand, in mRNA vaccines, mRNA that has been transcribed once is encapsulated in lipid nanoparticles and injected into the body. Then, the mRNA does not go to the nucleus, and it directly produces the spike protein in the cytoplasm. Additionally, the virus vector vaccine introduces the spike gene and infects the cells after being injected into the body. After being infected once, this weak virus transfers DNA to the nucleus, from which it becomes mRNA and peptides, presents antigens, and acquires immunity. Unlike DNA or mRNA vaccines, other viruses are used; if the virus originally has immunity, neutralizing antibodies will be present, and the effect will be attenuated.

Other common problems with these mRNA vaccines include temperature control, allergies, immunosuppressive effects, and risk of developing autoimmune diseases. For elderly people who are prone to severe illness, people with underlying diseases, and people who have no immunity to SARS-CoV-2 (such as those who have not been vaccinated), it is expected that the development of vaccines with both efficacy and safety will be an important issue. Furthermore, cross-vaccination, in which various vaccines are combined and vaccinated, is actually carried out. However, it is thought that the problem is that no clear criteria have been shown for specific vaccine combinations, such as vaccination intervals and the risk of adverse reactions. Additionally, regarding the production and supply system of vaccines, it has been pointed out that it is necessary to deal with various issues related to the response to new modality vaccine manufacturing such as nucleic acid vaccines, a manufacturing system that includes: procurement, maintenance, and management of large-scale manufacturing equipment that conforms to GMP (Good Manufacturing Practice) for the mass production of vaccines; and securing manufacturing personnel, and development of supply chains such as storage and transportation. Among the most pressing technical challenges in the development of the SARS-CoV-2 vaccine, which is considered to be an issue of great importance, are: the development of semi-universal vaccines effective against several mutant strains; development of biomarkers to evaluate vaccine efficacy; construction of an evaluation system that enables optimization of vaccine dosage and administration in clinical trials; and subject recruitment for clinical trials.

In summary, at present, most humans have immunity to SARS-CoV-2. Considering that even once infected, the immune system weakens in a short period of time, and that it may continue to spread around the world in the future, it is essential to develop a COVID-19-preventive vaccine that can strongly induce immunity and maintain immunity for a long time. However, some of the SARS-CoV-2 virus variants that have emerged so far have acquired the ability to escape from neutralizing antibodies. In addition, SARS-CoV-2 may undergo genetic mutation due to its continued spread, so a vaccine with broad cross-reactivity that can respond to antigenic changes associated with mutation is required.
